# ^18^ F-FDG PET-CT during chemo-radiotherapy in patients with non-small cell lung cancer: the early metabolic response correlates with the delivered radiation dose

**DOI:** 10.1186/1748-717X-7-106

**Published:** 2012-07-10

**Authors:** Mariangela Massaccesi, Maria Lucia Calcagni, Maria Grazia Spitilli, Fabrizio Cocciolillo, Francesca Pelligrò, Lorenzo Bonomo, Vincenzo Valentini, Alessandro Giordano

**Affiliations:** 1 Institutes of Radiotherapy, Università Cattolica del Sacro Cuore, Rome, Italy; 2 Institutes of Radiology, Università Cattolica del Sacro Cuore, Rome, Italy; 3 Institutes of Nuclear Medicine, Università Cattolica del Sacro Cuore, Largo A. Gemelli, 8, 00168, Rome, Italy

**Keywords:** 18F-FDG PET-CT, NSCLC, Chemo-radiotherapy, Metabolic response

## Abstract

**Background:**

To evaluate the metabolic changes on ^18^ F-fluoro-2-deoxyglucose positron emission tomography integrated with computed tomography (^18^ F-FDG PET-CT) performed before, during and after concurrent chemo-radiotherapy in patients with locally advanced non-small cell lung cancer (NSCLC); to correlate the metabolic response with the delivered radiation dose and with the clinical outcome.

**Methods:**

Twenty-five NSCLC patients candidates for concurrent chemo-radiotherapy underwent ^18^ F-FDG PET-CT before treatment (pre-RT PET-CT), during the third week (during-RT PET-CT) of chemo-radiotherapy, and 4 weeks from the end of chemo-radiotherapy (post-RT PET-CT). The parameters evaluated were: the maximum standardized uptake value (SUVmax) of the primary tumor, the SUVmax of the lymph nodes, and the Metabolic Tumor Volume (MTV).

**Results:**

SUVmax of the tumor and MTV significantly (p=0.0001, p=0.002, respectively) decreased earlier during the third week of chemo-radiotherapy, with a further reduction 4 weeks from the end of treatment (p<0.0000, p<0.0002, respectively). SUVmax of lymph nodes showed a trend towards a reduction during chemo-radiotherapy (p=0.06) and decreased significantly (p=0.0006) at the end of treatment. There was a significant correlation (r=0.53, p=0.001) between SUVmax of the tumor measured at during-RT PET-CT and the total dose of radiotherapy reached at the moment of the scan. Disease progression free survival was significantly (p=0.01) longer in patients with complete metabolic response measured at post-RT PET-CT.

**Conclusions:**

In patients with locally advanced NSCLC, ^18^ F-FDG PET-CT performed during and after treatment allows early metabolic modifications to be detected, and for this SUVmax is the more sensitive parameter. Further studies are needed to investigate the correlation between the metabolic modifications during therapy and the clinical outcome in order to optimize the therapeutic strategy. Since the metabolic activity during chemo-radiotherapy correlates with the cumulative dose of fractionated radiotherapy delivered at the moment of the scan, special attention should be paid to methodological aspects, such as the radiation dose reached at the time of PET.

## Background

About one third of patients with non-small cell lung cancer (NSCLC) present loco-regionally advanced disease at the diagnosis [1,2], and despite radical treatment with concurrent chemo-radiotherapy (chemo-RT), only 15% of patients will be long-term survivors and 15%–40% will develop loco-regional tumor recurrence [3,4]. A higher biologically effective dose of radiotherapy can improve loco-regional control and survival [5]: however, an escalating radiotherapy dose also results in increasing the risk of toxicity [6]. For this reason, it is important to carefully select patients for radiotherapy dose intensification. Currently, the response to radiotherapy is not determined until the therapy has been completed. If the individual response to radiotherapy could be evaluated earlier during treatment, a timely therapy modification could be accomplished to better adapt the cure. Molecular imaging offers the potential to characterize the nature of tissues on the basis of its biochemical and biologic features. ^18^ F-fluoro-2-deoxyglucose (^18^ F-FDG) positron emission tomography integrated with computed tomography (^18^ F-FDG PET-CT) is largely used in oncology, especially for monitoring the response to treatment. The imaging of changes in glucose metabolism, as reflected by cellular uptake and trapping of ^18^ F-FDG, can provide a response assessment that is both more timely and more accurate than that provided by standard morphological imaging [7]. Furthermore, the residual metabolic activity of tumors after radiotherapy, as measured by ^18^ F-FDG uptake, has been shown to correlate with the pathologic response [8], and to be a significant prognostic factor for survival in patients with NSCLC [9-11]. Many researchers recommend a delay of 6–8 weeks or longer after radiotherapy before performing the post-treatment PET study because of inflammatory changes with subsequent alterations in ^18^ F-FDG uptake [12]. Nevertheless, the confounding effect in the surrounding normal tissue due to the radiation-induced elevation of ^18^ F-FDG activity in the lung seems to be less relevant when PET is performed during radiotherapy [13]. The objectives of this study were: to evaluate the metabolic changes on serial ^18^ F-FDG PET-CT studies performed before, during and after concurrent chemo-radiotherapy in patients with unresectable or locally advanced NSCLC; to correlate the metabolic changes with the delivered radiation dose and with the clinical outcome.

## Methods

### Study population

Forty-three patients with unresectable or locally advanced NSCLC who were referred to our department from December 2005 to May 2008 were enrolled in this study. Eligibility criteria were good performance status (ECOG-performance status of 0 or 1), and a reasonable lung function (a forced expiratory volume in the 1^st^ second >50% of predicted value and a diffusing capacity of the lung for carbon monoxide >50%). Patients were not eligible if they had any other concomitant malignant disease, uncontrolled diabetes mellitus, or severe cardiac or cerebral diseases. Patients having undergone previous radiotherapy to the chest were not allowed to participate while those previously submitted to chemotherapy were accepted. Herein we describe only 25/43 patients (58%, 21 males and 4 females, mean age 64 years, range 43–78 years) who satisfied the eligibility criteria.

### Treatment description

All patients underwent concurrent chemo-RT. In the case of previous chemotherapy, concurrent chemo-RT was started after a minimum of 30 days from the last chemotherapy course. Radiotherapy was administered to the involved field with a three-dimensional conformal technique. The median International Commission on Radiation Units and Measurements (ICRU) total referred dose was 50.4 Gy with classical (1.8 Gy/day) fractionation. The planned target volume (PTV) consisted of the primary tumor and the gross nodal volume. Elective nodal irradiation was not administered. The treatment was planned with computed tomography, applying the lung parenchyma correctional factors. A linear photon accelerator (nominal energy 6–10 MeV) was used for the treatment in all cases. All patients were immobilized by customized devices. Two different concurrent chemotherapy regimens were used. Cisplatin (CDDP), 20 mg/m^2^/day/bolus during days 1–4 of the first and last week of treatment, plus weekly gemcitabine 350 mg/m^2^/day, was administered to patients who had not received previous chemotherapy. Two hundred and fifty mg/m^2^/day weekly gemcitabine alone was delivered to patients who had undergone prior chemotherapy. A radiological and pneumological re-assessment was performed 4 weeks from the end of chemo-radiotherapy. Patients judged operable underwent surgery, while those considered inoperable were treated according to the preference of the referring physician.

### ^18^ F-FDG PET-CT protocol: acquisition and reconstruction parameters, and image interpretation

Three ^18^ F-FDG PET-CT studies were performed using the same acquisition and reconstruction protocol: before starting chemo-RT (pre-RT PET-CT), during the third week of treatment (during-RT PET-CT), and 4 weeks from the end of treatment (post-RT PET-CT). The pre-RT PET-CT was performed at a median time of 13 days (range 1–29 days) before starting chemo-RT; the during-RT PET-CT was performed after a median time of 17 days from the start of treatment (range 10–25 days) and after the delivery of a median radiotherapy dose of 23.4 Gy (range 14.4-34.2 Gy); the post-RT PET-CT was performed at a median time of 30 days (range 16–36 days) from the end of treatment. In case of previous chemotherapy, at least one month had to pass between the last administration and the acquisition of the ^18^ F-FDG PET-CT. Details of the study were explained to the patients and they provided written informed consent as established by our ethics committee. All studies were performed using an integrated 3D PET-CT device (GEMINI GXL distributed by Philips Medical Systems) combining a dedicated full-ring PET scanner with gadolinium-oxyortho-silicate (GSO) crystals and a multislice spiral CT scanner. Prior to ^18^ F-FDG injection, patients fasting for at least six hours were settled in a quiet room, checked to be normoglycemic (patients with a fasting glucose level >150 mg/dl were excluded), and intravenously hydrated with saline solution (500 ml). No oral or intravenous contrast agents were administered or bowel preparation applied for any patient in our series. Images were acquired one hour after intravenous injection of 259–407 MBq of ^18^ F-FDG, produced in the radio-pharmacy of our Centre, according to the body mass index from pelvis to neck. The CT scan, performed from neck to pelvis with a voltage of 120 KeV and tube current of 30 mA, was used for the anatomical localization, and for attenuation correction of PET emission data. PET emission scans were acquired in 3D mode, from pelvis to neck (multiple bed positions, 3 minutes for each bed position). Matched CT and PET images were reconstructed with a field-of-view of 50 cm. Iterative reconstruction and CT-based attenuation correction were used and attenuation-corrected PET images were reviewed in transverse, sagittal and coronal planes. PET data were also displayed in a rotating maximum-intensity projection. To view the images, the PET and CT datasets were transferred to an independent computer workstation by DICOM (Digital Imaging and Communications in Medicine) transfer. PET-CT images were analyzed semi-quantitatively using the Syntegra Philips fusion program by two nuclear medicine physicians (M.L.C and M.G.S) with PET-CT experience. Regions of interest (ROIs) were manually drawn over the lesions (tumor and lymph nodes) showing an ^18^ F-FDG uptake higher than background activity. The Standardized Uptake Value (SUV) was measured in all voxels in the tumor ROI: SUV = (decay – corrected activity * ml tissue)/(activity injected * body weight). We used the maximum SUV (SUVmax) in order to minimize the partial volume effect. Therefore, we took into account the following parameters:

1. SUVmax of the tumor

2. Individual variation in SUVmax (ΔSUV) of the tumor, expressed as a percentage of the baseline

3. SUVmax of the lymph nodes

4. Individual variation in SUVmax (ΔSUV) of the lymph nodes, expressed as a percentage of the baseline value

5. Metabolic Tumor Volume (MTV), expressed in cc, obtained directly from the Philips workstation. Each tumor identified by the user was segmented automatically in three dimensions by the software using the following procedure. First, the voxel of maximum intensity along the selected projection line is used as the starting point for a region growing procedure. The algorithm then finds the voxel of local maximum intensity within a specified radius (default value of 1 cm) of the starting voxel. The region growing algorithm then defines the segmented volume as all voxels connected to the local maximum intensity voxel that have an intensity greater than a specified fraction of the maximum intensity. The threshold intensity value used in this study was 50% of the local maximum intensity. Once all of the hypermetabolic tumor foci are segmented, the software calculates the MTV, defined as the total volume of all tumors in the body in milliliters, as well as the maximum and average SUV within the MTV [14,15].

6. Individual variation in MTV (ΔMTV), expressed as a percentage of the baseline value

7. Metabolic response, according to the EORTC criteria [16], comparing the three PET-CT studies.

### Statistical analysis

The Systat 10.2 software (Systat inc., Point Richmond CA) was used for statistical analysis. Student’s paired *t*-test was used to compare the SUVmax and MTV at different time points. A p-value <0.05 was considered to indicate statistical significance. A linear regression analysis was performed to check out a possible relation between the metabolic response and the cumulative radiation doses reached in individual patients at the time of the during-RT PET-CT study. The disease-free survival (DFS; time to local or distant event) ‘time to event’ curve was calculated with the Kaplan–Meier method and statistical significance of the difference was assessed with the log-rank test.

## Results

Table 1 reports the clinical characteristics of all patients. Pre-RT PET-CT was available in 25/25 patients. During-RT PET-CT was available in 17/25 patients and no information was available for 8 patients due to logistic (3 patients) or technical problems (2 patients), and medical reasons (3 patients: hyperglycemia and radiation-related esophagitis). Post-RT PET-CT results were available for 21/25 patients and no information was available for 4 patients due to technical problems (1 patient) or clinical reasons (3 patients: early death due to myocardial infarction for 1 patient, and pulmonary infection in 2 patients).

**Table 1:** Patient characteristics

**Patient**	**Sex**	**Age**	**Pathology**	**cTNM**	**Metabolic response (EORTC) on during-RT PET-CT**	**Metabolic response (EORTC) on post-RT PET-CT**	**Surgery**	**pTNM**
1	F	43	Adenocarcinoma	T3N2M0	SMD	PMR	yes	T3N1M0
2	M	53	Adenocarcinoma	T2N1M0	PMR	PMR	yes	T2N0M0
3	F	54	Adenocarcinoma	T4N0M0	PMR	PMR	yes	T1N0M0
4	M	59	SCC	T2N2M0	PMR	CMR	yes	T1N1M0
5	F	60	Adenocarcinoma	T4N2M0	PMR	PMR	yes	T1N0M0
6	M	60	SCC	T3N2M0	PMR	CMR	yes	T1N0M0
7	M	64	Large cell carcinoma	T2N1M0	PMR	CMR	yes	T0N2M0
8	M	65	SCC	T4N1M0	PMR	PMR	no	
9	F	77	Adenocarcinoma	T4N2M0	PMR	PMR	no	
10	M	78	SCC	T3N2M0	PMR	CMR	yes	T1N0M0
11	M	62	Adenocarcinoma	T4N2M0	SMD	PMR	no	
12	M	70	Adenocarcinoma	T4N3M0	PMR	PMR	no	
13	M	73	SCC	T3N2M0	PMR	PMR	no	
14	M	55	Adenocarcinoma	T4N0M0	SMD	†	no	
15	M	58	Adenocarcinoma	T2N2M0	No scan	CMR	yes	T3N2M0
16	M	62	Large cell carcinoma	T4N2M0	PMR	No scan	yes	T3N0M0
17	M	63	Adenocarcinoma	T4N2M0	No scan	CMR	yes	T0N0M0
18	M	69	Adenocarcinoma	T3N2M0	No scan	CMR	yes	T0N0M0
19	M	71	SCC	T2N1M0	No scan	PMR	no	
20	M	62	SCC	T4N2M0	No scan	PMR	no	
21	M	69	SCC	T4N2M0	SMD	†	no	
22	M	68	SCC	T2N0M0	PMR	†	no	
23	M	74	Adenocarcinoma	T4N3M0	No scan	PMR	no	
24	M	63	Adenocarcinoma	T3N2M0	No scan	SMD	no	
25	M	65	SCC	T3N0M0	No scan	CMR	yes	T1N0M0

SCC, squamous cell carcinoma; CMR, complete metabolic response; PMR, partial metabolic response; SMD, stable metabolic disease. †Patient died within 70 days after radiotherapy.

### ^18^ F-FDG metabolic changes

**Figure 1:**
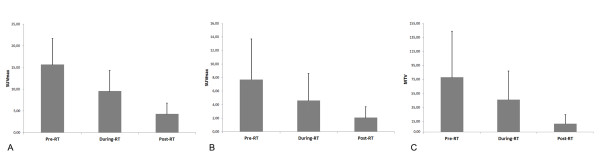
Mean value (±SD) of the SUVmax in the tumor (A) and lymph nodes (B), and the MTV (C) at different time-points in all patients (n = 25).

Figure 1 reports the mean value (±SD) of the SUVmax in the tumor (A) and lymph nodes (B), and the MTV (C) at different time-points. At pre-RT PET-CT, the mean value of SUVmax of the tumor was 16.1 ± 6.0 with a significant (p = 0.0001) reduction at during-RT PET-CT (10 ± 4.6). At post-RT PET-CT, the mean value of SUVmax of the tumor was 4.7 ± 2.6, a significant reduction when compared with either pre-RT PET-CT or during-RT PET-CT values: p = 0.0000, p = 0.005, respectively. At pre-RT PET-CT, the mean value of SUVmax of the lymph nodes was 7.7 ± 6.0, decreasing to 4.6 ± 4.0 at during-RT PET-CT (p = 0.06). At post-RT PET-CT, the SUVmax of the lymph nodes was 2.1 ± 1.6 showing a significant reduction when compared with either pre-RT PET-CT or during-RT PET-CT values: p < 0.0006, p = 0.02, respectively. At pre-RT PET-CT, the mean value of MTV was 78.6 ± 65.6 cc with a significant (p = 0.002) reduction at during-RT PET-CT (46.4 ± 40.8 cc). At post-RT PET-CT the MTV was 12 ± 13.1 cc showing a significant decrease when compared with either pre-RT PET-CT or during-RT PET-CT values: p = 0.0002, p = 0.002, respectively.

**Figure 2:**
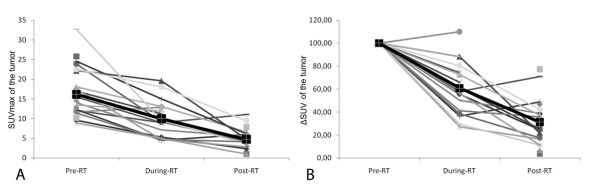
**SUVmax (A) and ΔSUV (B, expressed as a percentage of the baseline value) values of the primary tumor detected in all three**^**18**^ **F-FDG studies in all patients.** The black line shows the mean SUVmax of the group.

**Figure 3:**
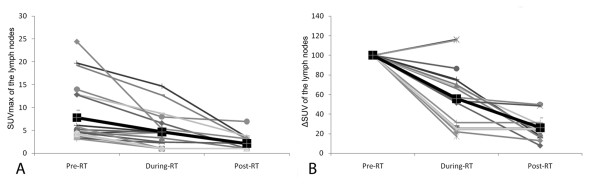
**SUVmax (A) and ΔSUV (B, expressed as a percentage of the baseline value) values of the lymph nodes detected in all three**^**18**^ **F-FDG studies in all patients.** The black line shows the mean SUVmax of the group.

**Figure 4:**
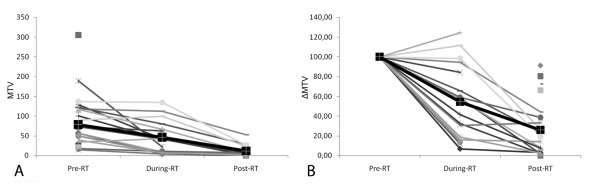
**MTV (A) and ΔMTV (B, expressed as a percentage of the baseline value) values of the primary tumor detected in all three**^**18**^ **F-FDG studies in all patients.** The black line shows the mean MTVmax of the group.

Figure 2 (A, B) reports the values of SUVmax and ΔSUV of the tumors measured in all three ^18^ F-FDG studies at different time points in all patients; Figure 3 (A, B) reports the values of SUVmax and ΔSUV in the lymph nodes measured in all three ^18^ F-FDG studies in all patients; Figure 4 (A, B) reports the values of MTV and ΔMTV in the tumors measured in the three ^18^ F-FDG studies in all patients. A large heterogeneity in SUVmax, in ΔSUV, in MTV, and in ΔMTV values was observed among patients in the tumors, as well as in the lymph nodes before, during and after chemo-radiotherapy.

**Figure 5:**
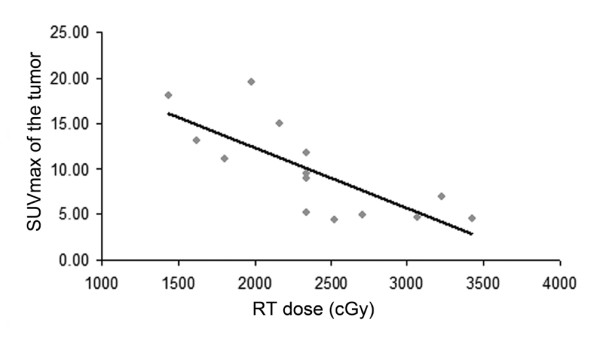
**Correlation (r = 0.53, p = 0.001) between the SUVmax of the primary tumor obtained at**^**18**^ **F-FDG during-RT and the total dose of radiotherapy reached at the moment of PET.**

Figure 5 reports a correlation between SUVmax of the tumor measured at during-RT PET-CT and the total dose of radiotherapy reached at the moment of the scan. A significant correlation (r = 0.53, p = 0.001) was found between SUVmax and the dose of radiotherapy delivered.

**Figure 6:**
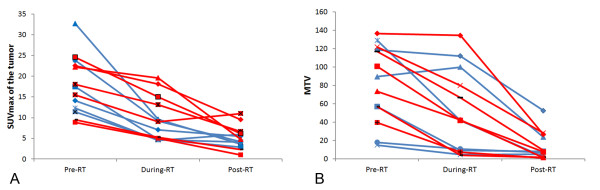
**SUVmax (A) and MTV (B) values of the primary tumor detected in all three**^**18**^ **F-FDG studies in responders (n = 6, blue lines) and non-responders (n = 7, red lines) patients.**

**Figure 7:**
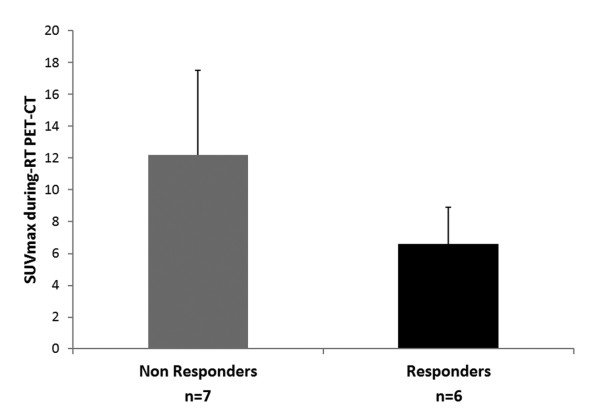
Mean value (±SD) of the SUVmax in the primary tumor measured at during-RT PET-CT in responders (n = 6) and non-reponders (n = 7) patients.

When applying the EORTC criteria [16] at during-RT PET-CT, 13/17 patients (76.5%) showed a partial metabolic response, and 4/17 patients (23.5%) a stable disease; no complete metabolic response was observed. When applying the EORTC criteria at post-RT PET-CT, 8/21 patients (38%) showed a complete metabolic response, 12/21 patients (57%) a partial metabolic response, and one patient (5%) showed stable disease (Table 1). When analyzing only the subset of patients for whom all three PET-CT were available (n = 13), we observed that the metabolic modifications were similar to those obtained in all series (see Table 1, Figure 1 and Figure 6): SUVmax of the tumor and of the lymph nodes, as well as the MTV, significantly decreased during and at the end of treatment. A borderline statistically significant difference (p = 0.05) between non-responders (n = 7; 12.2 ±5.9) and responders (n = 6; 6.6 ± 2.3) was found only in SUVmax of the tumor measured at during-RT PET-CT (Figure 7). There was no significant correlation between SUVmax of the tumor measured at during-RT PET-CT and SUVmax measured at post-RT PET-CT, as well as between MTV of the tumor measured at during-RT PET-CT and MTV measured at post-RT PET-CT.

### Clinical outcome and follow-up

Three out of twenty-five patients (12%) died: one patient during chemo-RT because of pulmonary infection, before completing the treatment; two patients died before undergoing post-RT PET-CT because of myocardial infarction and pulmonary infection. Thirteen out of twenty-five patients (52%) underwent surgery after a median time of 55 days from the end of treatment: 9/13 (69%) had pathological down-staging to stage 0 (no residual disease) or stage I, while 4/13 patients (31%) showed persistent loco-regionally advanced disease at a hystopathological examination. Tumors in 9/25 patients (36%) were judged unresectable and treated according to the preference of the referring physician.

After a median follow-up from the start of chemo-radiotherapy of 24.7 months (range 7–28 months), 12/25 patients (48%) are still alive while 13/25 patients (52%) died with a median survival time of 14 months. Fifteen patients showed disease progression (7 locoregional failures, 4 distant metastases and 4 both loco-regional and distant failures) during follow-up. Median disease progression free survival time was 13 months.

### Metabolic response assessment and clinical outcome

**Figure 8:**
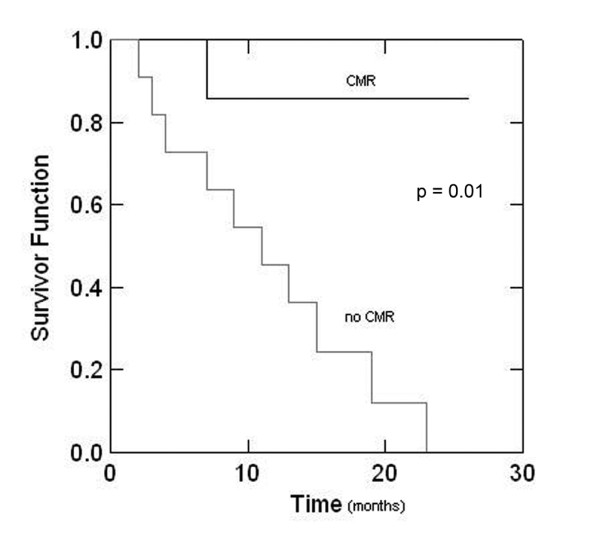
Disease progression free survival time according to metabolic response (EORTC criteria) after chemo-RT. CMR = complete metabolic response.

Figure 8 reports the disease progression free survival time in patients with complete metabolic response and in patients with no response at post-RT PET-CT, according to EORTC criteria. The disease progression free survival time was significantly longer (p = 0.01) in patients who showed a complete metabolic response than in patients with partial/stable metabolic response.

No other significant correlation with survival end-points (local and/or distant disease progression free survival and overall survival) was found for any other metabolic parameters evaluated at any different time-points. In patients who underwent surgery, no significant association was observed between pathologic down-staging and metabolic response.

## Discussion

^18^ F-FDG PET detects metabolic modifications which are well known to occur before morphologic ones; therefore functional imaging allows an evaluation of the tumor metabolic response during radiotherapy earlier than morphologic imaging [13,16,17]. Changes in therapy, such as dose-escalation or the addition of another treatment modality may be contemplated for patients who show poor response to the current radiotherapy regimen [18]. Furthermore, a disease reduction during radiotherapy detected by functional imaging might suggest a radiation dose escalation, whilst remaining within normal tissue constraints [19]. The role of ^18^ F-FDG in assessing the response to non-surgical treatment in patients with NSCLC has been extensively investigated. Many Authors performed an ^18^ F-FDG scan at least two months from the end of radiotherapy or chemotherapy [10,20-22], while only a few have evaluated the potential role of repeating ^18^ F-FDG PET earlier, either during or after therapy [13,17,23,24]. Researchers from the University of Michigan [13] have performed repeated ^18^ F-FDG PET in 15 patients with NSCLC stages I to III before, during and after a course of radiotherapy (or chemo-radiotherapy) with conventional fractionation. The Authors have observed a reduction of the peak tumor ^18^ F-FDG activity at approximately 45 Gy during radiotherapy, with a further reduction on PET performed three months from the end of treatment. The qualitative response during radiotherapy correlated with the overall response post-radiotherapy, and the peak tumor ^18^ F-FDG activity during radiotherapy correlated with that 3 months post-therapy. Subsequently, the same Authors [24] have observed that an adaptation of the radiotherapy plan based on ^18^ F-FDG PET at approximately 45 Gy during radiotherapy, might allow escalating the tumor dose without increasing the normal tissue complication probability. Van Baardwijk et al. [17] investigated changes in ^18^ F-FDG uptake in 23 patients with medically inoperable or advanced NSCLC who underwent serial ^18^ F-FDG PET-CT scans before treatment, 1 week and 2 weeks after the start of treatment and 70 days from the end of an accelerated (1.8 Gy twice a day) radiation treatment with radical intent. While 70 days after the end of radiotherapy, the metabolic activity of the tumor significantly decreased compared to the baseline, during the first 2 weeks of treatment the ^18^ F-FDG uptake within the tumor changed only moderately (a slight increase during the first week, and a slight decrease during the second week). More recently, Giovacchini et al. [25] performed four repeated ^18^ F-FDG PET-CT scans in 6 patients undergoing radical radiation treatment for either locally advanced or medically inoperable NSCLC. ^18^ F-FDG PET-CT scans were performed before, during radiotherapy at the delivered dose of 50 Gy, and after approximately one month and 3 months from the end of radiotherapy. Radiotherapy induced a progressive decrease in glucose metabolism that was greater 3 months after the end of treatment, but could even be detected during the treatment itself.

In this study, we have evaluated the metabolic changes on serial ^18^ F-FDG PET-CT performed before, during and after concurrent chemo-radiotherapy in patients with unresectable or locally advanced non-small-cell-lung-cancer (NSCLC). We have also correlated the metabolic changes with the delivered radiation dose, and with the clinical outcome. ^18^ F-FDG PET-CT studies were performed earlier both during treatment, at a median time of 17 days from the start (median dose of 23.4 Gy), and after treatment at a median time of 30 days. First of all, we observed that at pre-RT PET-CT the values of SUVmax of the tumors were much higher than those reported in literature [13,17], similar only to those reported by Giovacchini et al. [25]. The enhanced trapping of ^18^ F-FDG into the tumor cells can be due to either biological mechanisms, such as the up-regulation of glucose transporters and hexokinase enzymes, tumor aggressivity, hypoxia, etc., or to modifications induced by previous treatment [26-29]. Up to now, however, it is not known which of these mechanisms is responsible for the variable levels of ^18^ F-FDG uptake. In our study, the large tumor size, containing small areas of necrosis, and the relatively small number of patients who received previous treatment could explain the higher values of SUV at pre-RT PET-CT. From our data, we can observe that the tumor metabolic activity significantly decreased early during chemo-RT, and decreased even more at the end of treatment. The metabolic reduction was significant for all parameters, but more significant for the SUVmax. It can be argued that chemo-radiotherapy works better and faster in the cellular metabolism, when considering SUV values, and “relatively” less and more slowly in the tumor volume, based on MTV values. In fact MTV may be considered a “functional volume” and, as such, reduces its activity later in comparison with SUV. Therefore, SUVmax is the more sensitive parameter to show an earlier metabolic modification induced by the treatment. The difference in SUVmax reduction between pre-RT PET-CT and during-RT PET-CT was significantly higher (p = 0.0001) than that observed between during-RT PET-CT and post-RT PET-CT (p = 0.005). This interesting finding allow us to speculate that the tumor cells have a prompt response to treatment. On the contrary, the MTV reduction was similar between pre-RT PET-CT and during-RT PET-CT, as well as between during-RT PET-CT and post-RT PET-CT (p = 0.002, respectively), supporting the concept that MTV is a functional volume and its response to therapy is slower. Regarding the lymph nodes, their metabolic activity tends to decline during chemo-radiotherapy, decreasing significantly only at the end of treatment. This finding suggests the stronger lymph node resistance to therapy: in fact it is well known that the neoplastic lymph nodes are a negative prognostic factor especially in patients with NSCLC [30].

Finally, the tumor metabolic activity significantly decreased after a cumulative radiotherapy dose of only 23.4 Gy, which is much lower than that reported in literature: 45 Gy and 50 Gy [13,25]. On the other hand, van Baardwijk et al. [17] did not observe any significant decrease in tumor metabolic activity after the delivery of approximately 37 Gy of accelerated radiotherapy (1.8 Gy twice a day). Differences in the radiotherapy fractionation schedule, treatment time, concurrent chemotherapy administration, tumor biology, and absolute pre-RT PET-CT SUVmax values, might have an impact on tumor ^18^ F-FDG uptake during radiotherapy. Similarly to Kong et al. [13] and van Baardwijk et al. [17], we observed a large heterogeneity in the changes in metabolic activity among individual patients during and after radiotherapy: this finding may somehow reflect the difference in radio-responsiveness between the individual tumors. Similarly to these Authors [13,17], we also observed a large heterogeneity in the metabolic activity among the individual patients before treatment, suggesting a large cellular heterogeneity in each tumor: for this reason we have also utilized ΔSUV and ΔMTV in order to take into account the “individual” variations during and at the end of treatment, rather than only the “absolute” values such as SUVmax and MTV. In fact, the individual variations allow a better measurement of the effects of the treatment “normalizing” the baseline values, especially when they are highly heterogeneuos.

Regarding the metabolic response after treatment, the EORTC criteria [16] classifies the metabolic response on the basis of SUV values. The classification into four categories using only a number as cut-off, may sometimes give an incorrect classification. In fact, the number does not take into account some scintigraphic features, such as the distribution and shape of the ^18^ F-FDG activity. From our data, the high number of partial metabolic responses (>50%) can be also attributed to the use of strict SUV criteria, as proposed by EORTC. Therefore, from a clinical point of view, we strongly support the necessity to integrate the SUV values with qualitative and morphological (CT) analyses of the images since the confounding effects such as inflammation may be present at any time after treatment. A wrong classification might therefore be avoided and a clinical significance could be given to the ^18^ F-FDG uptake. Both Kong et al. [13] and van Baardwijk et al. [17], observed an association between tumor metabolic response during radiotherapy and that post treatment, with different patterns of response during radiotherapy for patients with a complete metabolic response, and patients with a persistence of metabolic activity after the therapy. Our results do not confirm these findings. We found a borderline statistical significant difference (p = 0.05) only in SUVmax of the tumor during treatment: non-responders showed higher value of SUVmax of the tumor at during-RT PET-CT when compared to responders patients. Unfortunately, our group sample is too small to speculate on this finding. Moreover the wide range in the cumulative radiation dose delivered until the moment of during-RT PET-CT acquisition (14.4-34.2 Gy) is likely to have contributed to the heterogeneity in metabolic response, and may represent a major drawback for this study. Indeed, we observed a significant correlation between SUVmax measured during-RT PET-CT and the cumulative dose of radiotherapy delivered at the moment of the scan acquisition. Although this finding is not surprising, and is in accordance with the well-known association between the radiation dose delivered and the probability of cure in NSCLC [31], this is the first time that it is clearly shown in a clinical setting. Further investigations of this association, i.e. describing tumor activity as a function of pre-treatment activity, radiotherapy dose delivered, and time since the beginning of radiotherapy, may prove to be very interesting. For example, dose-SUV curves could be elaborated using experimental data to extrapolate the total radiation dose required to obtain a complete metabolic response in each patient, thus making it possible to adapt the radiation dose prescription.

A significant correlation between the residual ^18^ F-FDG uptake within the tumor at the end of treatment (or change in ^18^ F-FDG uptake in respect to the baseline), and survival end-points has been described by several Authors [10,17]. Finally, also in our experience, patients with loco-regionally advanced NSCLC who showed a complete metabolic response at post-RT PET-CT had a longer disease-free survival when compared with those with a persisting ^18^ F-FDG uptake. The main limitation of this study is the small group of patients. More studies on a larger number of patients are necessary to confirm our findings.

## Conclusions

^18^ F-FDG PET-CT is able to detect earlier metabolic modifications in patients with locally advanced NSCLC who underwent pre-operative concurrent chemo-radiotherapy. SUVmax of the tumor is therefore a valuable parameter, much more so than MTV. While the metabolic changes of the tumor at the end of treatment seem to be of prognostic value for a progression disease-free survival, the metabolic changes during radiotherapy did not correlate either with the metabolic response after treatment or with the clinical outcome. However, we cannot conclude that ^18^ F-FDG PET-CT during chemo-radiotherapy does not provide any useful information. Indeed, our analysis has a major limitation, due to the wide range in radiation dose reached at the moment of the PET-CT scan, which may have impaired the interpretation of the results. Further studies exploiting the correlation between metabolic modifications during therapy and the clinical outcome are needed in order to optimize therapeutic strategy. Since the metabolic activity during chemo-radiotherapy correlates with the cumulative dose of radiotherapy delivered at the moment of the scan, special attention should be paid to methodological aspects, such as the radiation dose reached at the time of the PET scan.

## Abbreviations

18 F-FDG PET-CT, F-fluoro-2-deoxyglucose positron emission tomography integrated with computed tomography; Chemo-RT, chemo-radiotherapy; NSCLC, non small cell lung cancer; SUVmax, maximum Standardized Uptake Value; ΔSUV, individual variation of SUVmax expressed as a percentage of the baseline value; MTV, Metabolic Tumor Volume; ΔMTV, individual variation of MTV expressed as a percentage of the baseline value; EORTC, European Organization for Research and Treatment of Cancer.

## Competing interest

The authors declare no conflict of interest.

## Authors’ contributions

MM, MLC, VV, AG: conception and design. MM, MLC, MGS, MF acquisition of data, MM, MLC, FC analysis of data. MM, MLC alignment and drafted the manuscript. All authors read and approved the final manuscript.
